# Medical Items

**Published:** 1892-06

**Authors:** 


### *MEDICAL ITEMS.*

---

Dr. Luther B. Grandy has been elected Demonstrator of Anatomy in the Southern Medical College, to succeed Dr. W. S. Elkin, who becomes Professor of Operative and Clinical Surgery. The Southern College is now erecting its new building opposite the Grady Hospital, and especial pains will be expended to make the new dissecting room contain every facility for practical and thorough anatomical study.

---

Dr. M. B. Hutchins has been elected Demonstrator of Anatomy in the Atlanta Medical College, to succeed Dr. F. W. McRae, resigned. During the summer the anatomical room of the college will be enlarged to occupy one-half the whole upper floor, and the facilities for the study of practical anatomy will be greatly improved. Normal histology will also be taught by the Demonstrator. Dr. Hutchins will continue the practice of dermatology as heretofore, and his lectures at the college upon this subject.

---

In a recent paper Dr. W. A. Hammond has revived the question of castration as a mode of capital punishment. This is not new, as it was advocated more than a century and a half ago. Dr. Hammond thinks that the fear of being deprived of testicles would exercise a stronger deterring effect on the minds of criminals than the fear of death. Furthermore, the castrated individual might live to be of some use to society. He might make a good singer, or physician, or minister, and as a Pullman porter he would be invaluable.

Dr. Hammond's argument is stated in his usual pleasing and forcible manner, but, we doubt not, it will not meet with a very enthusiastic reception.

OUR esteemed contemporary, the *Texas Health Journal*, has a fearless way of dealing with frauds, and quacks, and never fails to give them a punch when the occasion presents. The last issue sounds the warning as to "Parker, the Nashville Quack, Dead-beat and Liar," whose "Lying" (in) advertisement has appeared a few times in different medical journals. It seems that Parker is a notorious blatherskite, who succeeded for a while in swindling both the lay and medical press. We promptly dismissed him from our columns as soon as we became apprised of his character and business.

Our contemporary mentions another, Lanphear, of Kansas City, connected with the "University Medical College" of that city. He is an itinerant surgeon, "going over the country, doing operations." According to the *Journal* Lanphear is a "rank and arrant quack" of the first water.

---

AT the last meeting of the American Surgical Association a movement was begun looking to the erection of a monument to the memory of Dr. S. D. Gross, in the city of Washington. Dr. Gross was probably, in his prime, the greatest surgeon in America and his useful life and labors are eminently deserving of a lasting memorial in our capital city. The profession and public at large are invited to assist. Dr. Jno. B. Roberts, 1627 Walnut street, Philadelphia, will receive and receipt all contributions.

---

TOUCHING the old question as to the ownership of the prescription, the *New York Herald* states that the druggist has no right claim to it. It is the property of the person prescribed for, or at least, of the person who employed the physician. The formula is the doctor's which he gives to the patient to make a temporary use of. The druggist has no right to either paper or ideas, and can only insist upon having a copy for his own protection.

AN appeal was recently made to the Prussian House of Representatives by the over-humane and tender-hearted "International and Hanoverian Association for the Suppression of the Scientific Torture of Animals," urging the absolute prohibition of vivisection. We are pleased to learn that the petition of the "I. H. A. S. S. T. A., etc.," was rejected, the committee on education reporting that science could not dispense with vivisection.

---

THE Legislature of Ohio, at the instance of the "physio-medics," recently refused to pass a measure to regulate the practice of medicine in the State, but appropriated $5,000 for the purpose of testing the value of the so-called cure for drunkenness. The Cincinnati *Lancet-Clinic* trusts "that a large number of the members of the Legislature will submit themselves as subjects for experiment."

---

DR. GŒLET states (*Archives of Gynecology and Obstetrics*) that the best urinary antiseptic is oil of wintergreen, given in doses of four to six drops in capsules four times a day. For the same purpose Gaubet prefers salol, half a drachm to a drachm and a half during tne day.

---

WE have tried to keep our readers informed as to all the new "cures" for phthisis. The latest is monochlorophenol, a volatile relative of that vile compound trichlorophenol. As usual, many cases have been completely cured, after one or two months' use of the drug.

---

THE Alumni of the Medical College of South Carolina (Charleston) have organized an association, and will establish and equip a laboratory of biology and pathology. Dr. Eugene Wasden, of the Marien Hospital Service, is president, and Dr. C. B. Calson, of Charleston, is secretary and treasurer.

---

A BRONZE statue of Dr. J. Marion Sims will soon be placed in position in Central Park, New York City.

